# Prevalence of musculoskeletal disorders among dental healthcare providers: A systematic review and meta-analysis

**DOI:** 10.12688/f1000research.124904.2

**Published:** 2022-12-12

**Authors:** Deepika Chenna, Kalyana C Pentapati, Mathangi Kumar, Medhini Madi, Hanan Siddiq

**Affiliations:** 1 Department of Immunohematology and Blood Transfusion, Kasturba Medical College, Manipal, Manipal Academy of Higher Education, Manipal, Karnataka, 576104, India; 2 Department of Public Health Dentistry, Manipal College of Dental Sciences, Manipal, Manipal Academy of Higher Education, Manipal, Karnataka, 576104, India; 3 Department of Oral Medicine and Radiology, Manipal College of Dental Sciences, Manipal, Manipal Academy of Higher Education, Manipal, Karnataka, 576104, India

**Keywords:** musculoskeletal disorders, workplace, dentist, dental students, dental auxiliary, systematic review

## Abstract

**Background**: Work-related musculoskeletal disorders (MSD) are common in dentistry due to the prolonged static work involved during patient care, making dental health care personnel vulnerable to musculoskeletal complaints. We aimed to pool the prevalence estimates of MSD among various dental healthcare providers, including dentists, dental students, dental hygienists, and auxiliaries.

**Methods**: A systematic search of five databases was performed (Scopus, Embase, CINAHL, Web of Science, Dentistry & Oral Sciences Source). The studies that reported the prevalence of MSD among dental healthcare workers and those written in English were selected. Screening and data extraction were performed by two review authors independently. Discrepencies were resolved by another review author. Risk of bias assessment was done using a nine-item questionnaire developed by Hoy *et al*. Pooled estimates were calculated using meta-analysis of proportions (random effects model).

**Results**: Among the 3090 publications screened, 234 publications were included for full-text screening. Meta-analysis was performed for 89 estimates from 88 publications. Females showed significantly higher prevalence [OR = 1.42 (95% CI = 1.09–1.84); I ^2 ^= 66.02; N = 32]. The analysis yielded a pooled estimate of 78.4% (95% CI = 74.8–82). The meta-regression showed similar prevalence over the years (Coefficient: 0.001; P-value: 0.762).

**Conclusions**: A high prevalence of MSD was noted among dental healthcare providers, with about seven out of ten having experienced MSD in the past. This emphasizes the need for awareness and adoption of appropriate ergonomic postures by dental healthcare providers from early in their careers to minimize work-related MSD.

## Introduction

“Musculoskeletal disorders (MSD) are injuries to the human support system of muscles, ligaments, tendons, nerves, blood vessels, bones, and joints” ( https://www.cdc.gov/). MSDs are defined as musculoskeletal system and connective tissue diseases and disorders when the event or exposure leading to the case is bodily reaction (e.g., bending, climbing, crawling, reaching, twisting), overexertion, or repetitive motion. MSDs do not include disorders caused by slips, trips, falls, or similar incidents (Bureau of Labor Statistics of the Department of Labor. NIOSH workers health chartbook 2004. NIOSH Publication No. 2004-146. Washington, D.C). Such injuries resulting due to occupation or work-related exposure are termed work-related MSD. Work-related MSD is common in dentistry due to the prolonged static work involved during patient care, making dental health care personnel vulnerable to musculoskeletal complaints. Moreover, the current lifestyle practices make the onset of such problems likely at an early stage of life. MSD includes pain, discomfort, or limitation in a range of activities in the head, neck, shoulders, arms, wrists, fingers, elbows, upper and lower back, buttocks, thighs, feet, ankle, etc.

MSD among dental healthcare personnel can potentially impact the individual and the community. Literature has shown a decrease in work efficiency, stress, poor sleep quality, multisite pain, frequent absenteeism, and/or early retirement resulting in loss of workforce. ^1^^,^^2^ The preventive strategies adopted to mitigate MSD are massage treatments, increased physical activity, adopting ergonomically designed equipment, maintaining correct postures, and using complementary and alternative medicine. ^3^^,^^4^

The studies on self-reported MSD have reported a high prevalence among dental healthcare personnel. ^5^^–^^10^ Studies have also evaluated the associated risk factors of MSD among dentists, ^7^^,^^11^^–^^19^ dental hygienists, ^6^^,^^20^^,^^21^ and dental students. ^22^^,^^23^ Increasing age, gender (female), comorbidities, prolonged working hours, increased patient load, lack of physical exercise, non-usage of loupes, stress, lack of breaks between patients, awkward postures, administrative work, vibration, and repetition were some of the reported risk factors of MSD. ^4^^,^^24^ A few literature reviews and meta-analysis on these conditions have reported a high prevalence among dental healthcare personnel. ^25^^–^^31^ However, there was no attempt to study the overall prevalence estimates of MSD burden among various dental healthcare providers, including dentists, dental students, dental assistants, and auxiliaries at a global level. Hence, we aimed to pool the estimates of the MSD burden among dental healthcare providers.

## Methods

### Inclusion and exclusion criteria

The studies that reported the overall prevalence of MSD among dental healthcare personnel (dentists, dental students, hygienists, or dental auxiliaries), and the studies written in English were included. Only cross-sectional studies and cohort studies, where prevalence data can be extracted or calculated were included. The studies reported as commentaries, letters, or conference abstracts were excluded. The protocol was registered with INPLASY ( DOI: 10.37766/inplasy2021.5.0100). ^32^

### Literature search

A systematic search in five databases (Scopus (RRID:SCR_022706), Embase (RRID:SCR_001650), CINAHL (RRID:SCR_022707), Web of Science (RRID:SCR_022706), Dentistry & Oral Sciences Source (RRID:SCR_022705)) from inception to 5 August 2021 was performed. The keywords used were “dentist OR dental hygienist OR dental personnel OR dental student” AND “musculoskeletal disease OR musculoskeletal disorder OR occupational disease OR work-related musculoskeletal disorder.” Suitable filters (reports on humans, research articles) for each database were applied.

### Screening

The search was imported to Rayyan, a web-based application (RRID:SCR_017584). ^33^ The screening and data extraction were done by two review authors independently (MK and MM). Disagreements were arbitrated by another review author (PKC). Agreement between the reviewers for title and abstract screening and full text screening showed almost perfect agreement (Kappa: 0.94 and 0.98 respectively).

***Risk of bias (RoB) assessment***

All studies were assessed using the 10 item Quality Assessment Checklist for Prevalence Studies questionnaire ^34^ by two review authors independently (HS and PKC). Disagreements were arbitrated by another review author (CD). Each question has two levels, low risk (0) and high risk (1). The total of all nine questions was used to categorize the studies as “low (0–3), moderate (4–6), or high risk (7–9)”.

### Data extraction

The variables for data extraction included study details such as authors, year, country, continent, study design, sample size, type of participants (dentist or dental students, or dental auxiliaries), age distribution, sex distribution, the overall prevalence of MSD at maximum recall along with lifetime, annual, one-week prevalence, gender and site-specific estimates.

### Statistical analysis

Due to variation in the reporting of the prevalence of MSD among the included studies, the prevalence estimates at the maximal follow-up were used to calculate the pooled estimates of MSD. Measures of heterogeneity (Q and I ^2^) were calculated. A random-effects model (restricted maximum likelihood estimation method) was used to calculate the prevalence estimates using the OpenMeta[Analyst] software for Windows 8 (Metafor Package 1.4, 1999) (RRID: SCR_022698). Time trends of MSD were evaluated using meta-regression. A sub-group analysis based on the continent, country, type of dental personnel, site of MSD, and sex was performed. A funnel plot was used to evaluate the publication bias. Complete data for the analysis can be accessed at Mendeley datasets. ^35^

## Results

**Figure 1.**
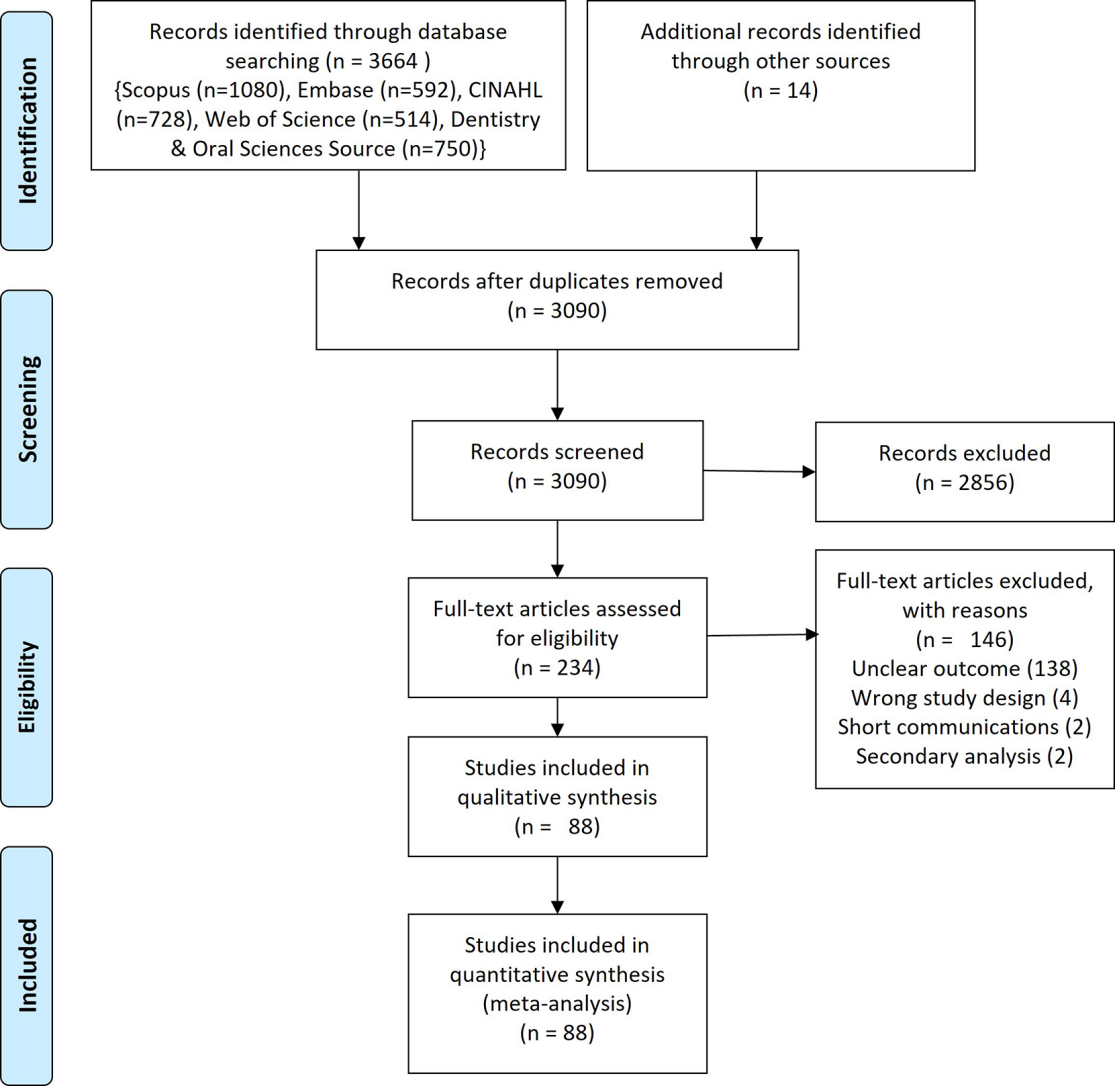
PRISMA flow chart.

A comprehensive systematic search of five databases (Scopus (1080), Embase (592), CINAHL (728), Web of Science (514), Dentistry & Oral Sciences Source (750)) yielded a total of 3664 articles. Reviews, conference proceedings, case reports, clinical trials, studies on ergonomics, quality of life, burnout, etc. letters, magazine reports, work related hazards other than MSD, studies among health professionals other than dentists were excluded (n = 2856). A further 146 publications were excluded after screening the full-text. Meta-analysis was performed for 89 estimates ( Table 1 and Figure 1).

**Table 1.** Characteristics of the included studies in the meta-analysis.

Author and year	Continent	Sample size	Population	ROB	Prevalence
Osborn *et al*. 1990	NA	385	Dentists	Low	68.31
Rundcrantz *et al*. (a) 1990	Eu	311	DA	Low	83.28
Rundcrantz *et al*. (b) 1991	Eu	311	Dentists	Low	84.24
Marshall *et al*. 1997	Au	355	Dentists	Low	81.97
Akesson *et al*. 1999	Eu	74	ALL	Low	91.89
Kerosuo *et al*. 2000	Eu	228	Dentists	Low	70.61
Lalumandier *et al*. 2001	NA	5119	ALL	Low	47.14
Anton *et al*. 2002	NA	95	DA	Low	92.63
Szymanska 2002	Eu	268	Dentists	Low	91.42
Tezel *et al*. 2005	Asia	221	DS	Low	85.97
Leggat *et al*. 2006	Au	285	Dentists	Low	87.37
Polat *et al*. 2007	Asia	120	Dentists	Low	94.17
Puriene *et al*. 2008	Eu	1670	Dentists	Low	86.53
de Carvalho *et al*. 2009	SA	227	DS	Low	70.93
Akar *et al*. 2009	Asia	185	DA	Low	23.78
Ayers *et al*. 2009	Au	560	Dentists	Low	59.82
Dajpratham *et al*. 2010	Asia	163	ALL	Low	96.93
Kierklo *et al*. 2011	Eu	220	Dentists	Low	90.00
Ellapen *et al* 2011	Africa	94	Dentists	Low	54.26
Moradia and Prakash 2011	Asia	77	ALL	Low	63.64
Sankar *et al*. 2012	Asia	259	Dentists	Low	41.70
Tzu *et al*. 2012	Asia	197	Dentists	Low	92.39
Muralidharan *et al*. 2013	Asia	73	Dentists	Low	78.08
Kumar *et al*. 2013	Asia	536	Dentists	Low	100.00
Vuletic *et al*. 2013	Eu	89	Dentists	Low	69.66
Kazancioglu *et al*. 2013	Asia	608	Dentists	Low	87.01
Rafeemanesh *et al*. 2013	Asia	58	Dentists	Low	82.76
Zoidaki *et al*. 2013	Eu	80	Dentists	Low	82.50
Movahhed *et al*. 2013	Asia	177	DS	Low	83.62
Sustova *et al*. 2013	Eu	182	DS	Low	39.01
Vora *et al*. 2014	Asia	86	Dentists	Low	62.79
Zarra and Lambrianidis 2014	Eu	120	Dentists	Low	60.83
Mendegeri *et al*. 2014	Asia	60	Dentists	Low	88.33
Shadmehr *et al*. 2014	Asia	446	Dentists	Low	80.94
Kursun *et al*. 2014	Asia	264	DS	Low	48.48
Tirgar *et al*. 2015	Asia	60	Dentists	Low	93.33
Gupta *et al*. (a) 2015	Asia	877	Dentists	Low	71.04
Humann *et al*. 2015	NA	488	DA	Low	98.36
Sakzewski *et al*. 2015	Au	466	Dentists	Low	86.05
Kanaparthy *et al*. 2015	Asia	134	DS	Moderate	53.73
Aljanakh *et al*. 2015	Asia	68	Dentists	Low	77.94
Alghadir *et al*. 2015	Asia	146	Dentists	Low	84.93
Hodacova *et al*. 2015	Eu	575	Dentists	Low	97.91
Bhagwat *et al*. 2015	Asia	200	Dentists	Low	57.50
Gupta *et al*. (b) 2015	Asia	2879	Dentists	Low	100.00
Sahu *et al*. 2015	Asia	206	Dentists	Low	81.07
Tamo *et al*. 2015	Asia	156	Dentists	Low	70.51
Batham and Yasobant 2016	Asia	93	Dentists	Low	92.47
Rehman *et al*. 2016	Asia	120	DS	Low	70.00
Kriangkrai *et al*. 2016	Asia	68	DS	Low	100.00
Rayyan *et al*. 2016	Asia	191	DS	Low	83.77
Cho *et al*. 2016	Asia	401	Dentists	Low	86.78
Phedy *et al*. 2016	Asia	241	Dentists	Low	63.49
Freire *et al*. 2016	SA	94	Dentists	Low	90.43
Al-Rawi *et al*. 2016	Asia	101	Dentists	Low	67.33
Barry *et al*. 2017	NA	337	DA	Low	80.42
Garbin *et al*. 2017	SA	204	Dentists	Low	81.37
Taib *et al*. 2017	Asia	82	Dentists	Low	100.00
Al-Hourani *et al*. 2017	Asia	81	DA	Low	100.00
Revankar *et al*. 2017	Asia	150	Dentists	Moderate	81.33
Hegde *et al*. 2018	Asia	200	Dentists	Low	97.00
Hosseini *et al*. 2019	Asia	136	Dentists	Low	91.91
Scepanovic *et al*. 2019	Eu	87	ALL	Low	79.31
El Naji *et al*. 2019	Asia	134	Dentists	Low	19.40
Benlidayi *et al*. 2019	Asia	99	DS	Low	85.86
Zafar *et al*. 2019	Asia	142	DS	Low	58.45
dos Santos *et al*. 2019	SA	241	DS	Low	82.57
Meisha *et al*. 2019	Asia	234	Dentists	Low	70.09
Gandham *et al*. 2019	Asia	150	Dentists	Low	58.67
Khandan *et al*. 2020	Asia	51	Dentists	Low	84.31
Netanely *et al*. 2020	Asia	102	DA	Low	89.22
Harris *et al*. 2020	NA	647	DA	Low	82.53
Pope-Ford *et al*. 2020	NA	14	Dentists	Moderate	92.86
Senosy *et al*. 2020	Asia	66	Dentists	Low	89.39
Shekhawat *et al*. 2020	Asia	72	Dentists	Low	100.00
Rahman *et al*. 2020	Asia	82	DA	Low	81.71
Uppada *et al*. (b) 2020	Asia	624	Dentists	Low	69.07
Aboalshamat 2020	Asia	332	ALL	Low	81.33
Ohlendorf *et al*. (b) 2020	Eu	450	ALL	Low	95.78
Ohlendorf *et al*. (a) 2020	Eu	406	DA	Low	98.52
Uppada *et al*. (a) 2020	Asia	156	Dentists	Low	84.62
Kumar M *et al*. 2020	Asia	151	ALL	Low	58.28
Berdouses *et al*. 2020	Eu	1500	Dentists	Low	54.07
Ahmad *et al*. 2020	Asia	244	Dentists	Low	86.48
Hashim *et al*. 2021	Asia	202	DS	Moderate	68.32
Alnaser *et al*. 2021	Asia	186	Dentists	Low	47.85
Gandolfi *et al*. 2021	Eu	284	ALL	Low	84.86
Felemban *et al*. 2021	Asia	377	DS	Low	91.25
Bhuvaneshwari *et al*. 2021	Asia	545	Dentists	Low	88.07

Eu: Europe; NA: North America; SA: South America; Au: Australia; DA: Dental auxillaries; DS: Dental students; ALL: All types of dental health care personnel; ROB: Risk of Bias.

### Prevalence

The prevalence of MSD ranged from 19.4 to 100%. Only seven publications showed less than 50% of MSD. ^17^^,^^36^^–^^41^ More than one-quarter (n = 24) of the included publications reported more than 90% prevalence. ^5^^–^^12^^,^^16^^,^^18^^,^^23^^,^^42^^–^^54^ One fourth of the studies (n = 21) reported a lifetime prevalence, ^3^^,^^37^^,^^39^^,^^44^^,^^45^^,^^49^^,^^51^^,^^53^^–^^66^ while only eight studies reported a one-week prevalence. ^8^^,^^18^^,^^19^^,^^22^^,^^42^^,^^53^^,^^54^^,^^67^ Most of the included studies reported a one-year prevalence (n = 65) ( Table 1).

### Age

Most of the studies reported the age distribution of the participants (n = 61), while 14 studies reported only the age range of the participants. Prevalence estimates could not be calculated as there was substantial variation in age grouping.

### Gender

**Figure 2.**
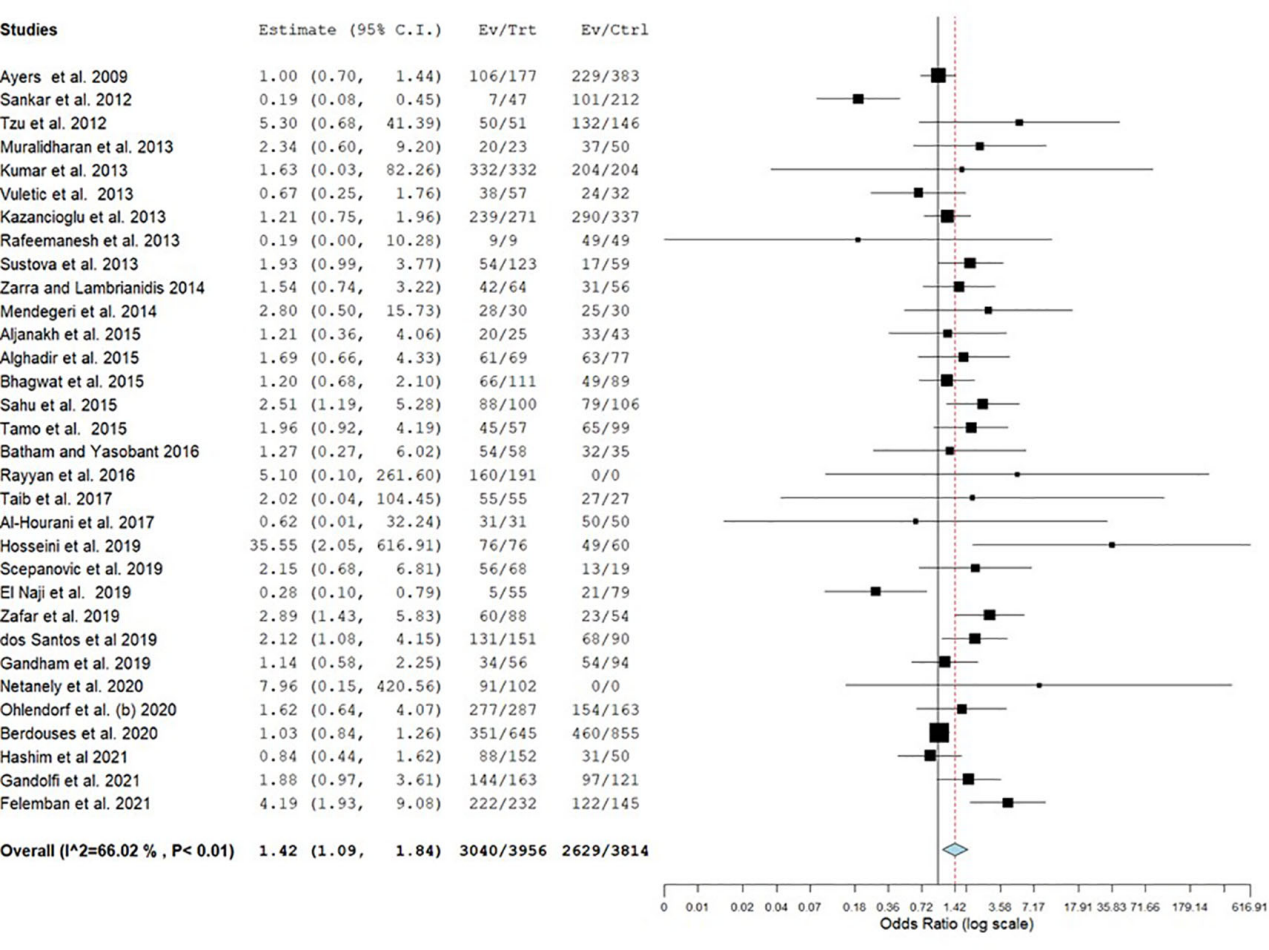
Forest plot of gender difference in the prevalence of musculoskeletal disorders (MSD).

Most of the studies reported the gender distribution of the participants (n = 80). Only one-third of the studies (n = 32) reported gender-specific estimates. The pooled prevalence of MSD among males and females was 72.4% (95% CI = 65.2–79.6) and 77.4% (95% CI = 69.4–85.4) respectively ^6^^,^^7^^,^^10^^,^^12^^,^^13^^,^^16^^,^^18^^,^^22^^,^^23^^,^^38^^,^^39^^,^^41^^,^^53^^,^^56^^,^^58^^,^^59^^,^^62^^,^^67^^–^^80^ ( Table 2). Females had significantly higher estimates of MSD than males (OR = 1.42) ( Figure 2).

**Table 2.** Sub-group analysis of the pooled estimates of overall musculoskeletal disorders.

Characteristic	Estimate (95% CI)	Q	I ^2^	N
Recall interval				
Overall	0.78 (0.75–0.82)	13941.24	99.82	89
Lifetime	0.78 (0.7–0.85)	4752.99	99.4	21
One year	0.82 (0.78–0.85)	4922.35	99.79	65
One week	0.66 (0.52–0.79)	330.09	97.67	8
Sex				
Male	0.73 (0.65–0.8)	1914.47	98.69	30
Female	0.77 (0.69–0.85)	2047.83	99.41	32
Dental personnel				
Dentists	0.79 (0.75–0.83)	5788.91	99.85	56
Dental auxiliaries	0.83 (0.69–0.97)	768.63	99.7	10
Dental students	0.73 (0.64–0.82)	671.93	98.09	14
Mixed	0.78 (0.66–0.89)	2346.13	99.16	9
Continent				
North America	0.8 (0.67–0.93)	3272.59	99.5	7
Europe	0.8 (0.72–0.88)	1530.95	99.29	17
Australia	0.79 (0.66–0.91)	124.04	97.79	4
Asia	0.78 (0.73–0.83)	4693.83	99.88	56
South America	0.81 (0.74–0.89)	21.15	87.39	4
Country				
US	0.8 (0.64–0.95)	3263.61	99.59	6
Sweden	0.86 (0.81–0.91)	5.485	67.53	3
Australia	0.85 (0.82–0.88)	3.984	49.84	3
Turkey	0.71 (0.48–0.94)	506.76	99.3	6
Brazil	0.81 (0.74–0.89)	21.15	87.39	4
India	0.77 (0.7–0.85)	1744.26	99.94	20
Iran	0.86 (0.82–0.91)	20.21	71.87	6
Greece	0.66 (0.49–0.82)	41.93	95.13	3
Saudi	0.76 (0.66–0.85)	133.73	95.64	8
Malaysia	0.9 (0.79–1)	43.86	94.73	3
Risk of bias				
Low	0.79 (0.75–0.82)	13700.42	99.83	85
Moderate	0.74 (0.58–0.9)	37.04	93.86	4

### Geographic distribution

Only a few studies were reported from North America (n = 7), ^37^^,^^43^^,^^49^^,^^52^^,^^63^^,^^81^^,^^82^ South America (n = 4), ^19^^,^^50^^,^^77^^,^^83^ and Australia (n = 4), ^14^^,^^68^^,^^84^^,^^85^ while only one study was reported from Africa. ^86^ Most of the studies were from Asia ^3^^,^^5^^–^^10^^,^^12^^,^^15^^–^^18^^,^^22^^,^^23^^,^^36^^,^^38^^,^^40^^,^^41^^,^^45^^,^^46^^,^^48^^,^^51^^,^^55^^–^^62^^,^^64^^–^^66^^,^^69^^–^^71^^,^^73^^–^^76^^,^^78^^,^^79^^,^^87^^–^^100^ and Europe ^11^^,^^13^^,^^39^^,^^42^^,^^44^^,^^47^^,^^53^^,^^54^^,^^67^^,^^72^^,^^80^^,^^101^^–^^105^ ( Table 2). Countries with more than three studies were included for the sub-group analysis. The highest pooled prevalence was seen in Malaysia, and the lowest pooled prevalence was seen in Greece.

### Risk of bias (RoB)

Out of the 88 studies included, only four studies had a moderate RoB. ^22^^,^^52^^,^^57^^,^^60^ The pooled estimates for studies with low and moderate RoB were 79% and 74% ( Table 2).

### Site distribution

The commonly reported sites were the neck, back, lower back, shoulder, upper back, and wrists. The least affected sites were thighs, legs, arms, feet, and ankles ( Table 3).

**Table 3.** Site-specific pooled estimates of overall musculoskeletal disorders.

Site	Estimate (95% CI)	Q	I ^2^	N
Neck	0.51 (0.46–0.56)	11158.95	98.86	78
Shoulder	0.41 (0.36–0.47)	8921.3	99.09	71
Wrist	0.31 (0.27–0.35)	4668.92	98.39	65
Arm	0.11 (0.07–0.15)	269.08	96.9	14
Elbow	0.16 (0.11–0.2)	1666.43	98.82	50
Fingers	0.18 (0.06–0.3)	260.81	98.23	6
Hip	0.16 (0.13–0.2)	1697.04	97.31	49
Thighs	0.1 (0.06–0.14)	92.07	89.32	10
Knee	0.18 (0.15–0.21)	1483.36	95.92	49
Leg	0.11 (0.06–0.17)	604.18	99.34	19
Ankle	0.14 (0.11–0.17)	1023.76	97.12	41
Feet	0.13 (0.06–0.2)	302.74	97.02	10
Back	0.5 (0.39–0.6)	8971.27	99.45	17
Lower back	0.46 (0.42–0.5)	3142.31	97.49	66
Upper back	0.35 (0.3–0.4)	3480.2	97.99	58

### Meta-analysis

**Figure 3.**
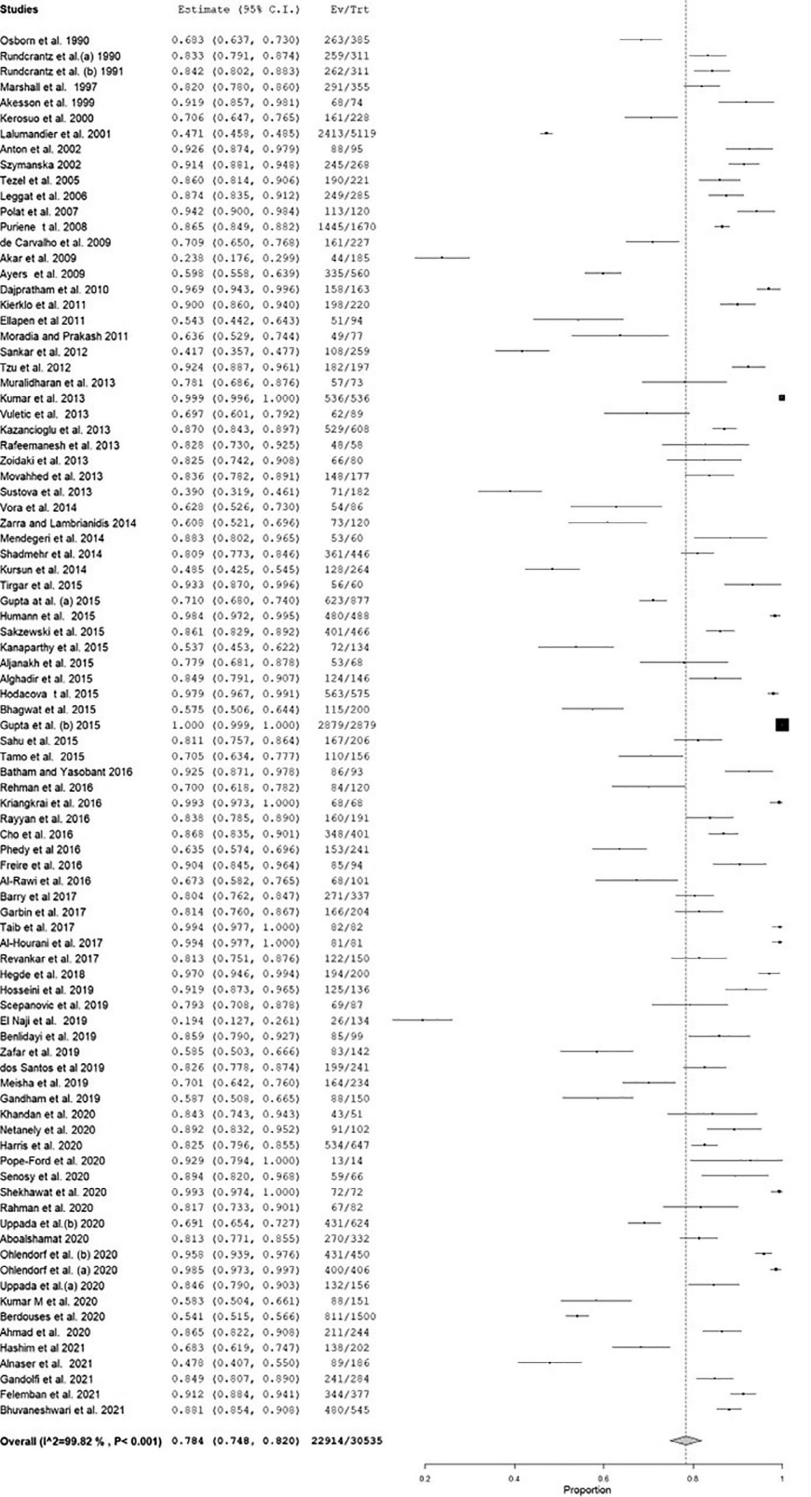
Forest plot of the prevalence of musculoskeletal disorders (MSD).

**Figure 4.**
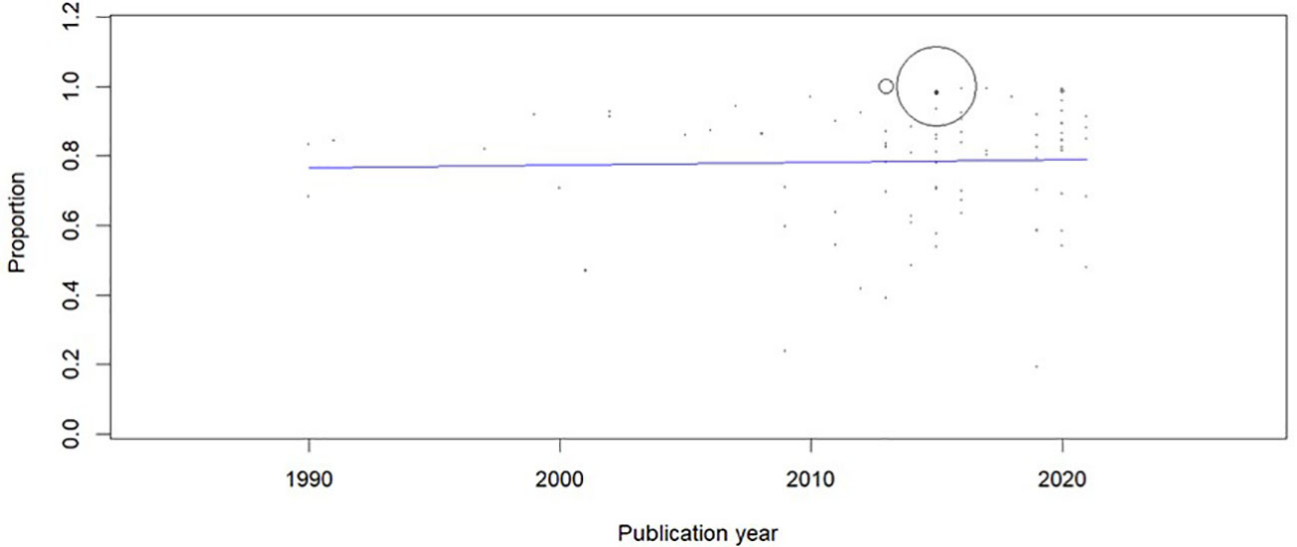
Meta-regression to evaluate the trends in the prevalence of musculoskeletal disorders (MSD).

**Figure 5.**
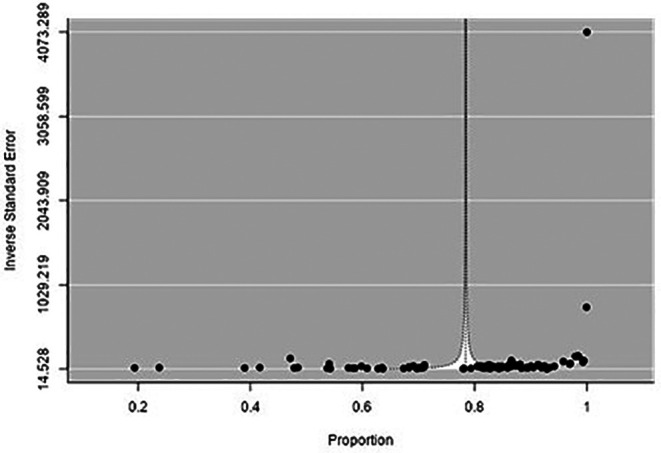
Funnel plot to evaluate the publication bias.

There was high heterogeneity among the included studies, as evidenced by Q and I ^2^ statistics. The model yielded a pooled estimate of 78.4% ( Figure 3), and sensitivity analysis did not show any change in the overall estimate. The meta-regression showed no change in the trend of MSD (Coefficient: 0.001; 95% CI: -0.004 to 0.006) ( Figure 4). Asymmetry was noted in the funnel plot (p < 0.001) ( Figure 5).

## Discussion

MSD’s result in pain, discomfort, or limitation in the range of movement. They are preventable conditions often due to poor ergonomic postures adopted by dental health care providers. We aimed to pool the estimates of MSD among dental healthcare providers. Eighty-eight publications recorded a comprehensive assessment of all body areas and reported the overall prevalence of MSD. The estimates needed to be evaluated carefully due to the high heterogeneity. The overall estimate was 78%, which was much higher than Greek and Czech surveys. ^37^^,^^80^ However, extensive surveys of dentists from India and Lithuania have reported similar or higher prevalence estimates. ^3^^,^^9^^,^^101^ Therefore, it is clear that dental professionals have quite a higher prevalence of MSD. Age-specific prevalence estimates could not be estimated due to a lack of standardized age groups or specific prevalence estimates. It was found that females showed higher prevalence estimates than males. Although the number of studies that reported gender distribution was high, only one-third of these studies reported gender-specific estimates of MSD.

The prevalence estimates were similar across the continents. The highest number of studies were reported from the Asian continent. The highest number of studies were from India, ^3^^,^^5^^,^^9^^,^^10^^,^^18^^,^^38^^,^^51^^,^^55^^,^^56^^,^^59^^,^^60^^,^^64^^,^^65^^,^^69^^,^^74^^,^^75^^,^^78^^,^^88^^,^^97^^,^^99^ followed by the US, ^37^^,^^43^^,^^49^^,^^52^^,^^81^^,^^82^ Iran, ^15^^,^^16^^,^^48^^,^^66^^,^^71^^,^^89^ and Turkey. ^36^^,^^40^^,^^45^^,^^61^^,^^70^^,^^87^ Studies from Malaysia ^7^^,^^95^^,^^98^ reported the highest prevalence estimates among various countries, followed by Iran, ^15^^,^^16^^,^^48^^,^^66^^,^^71^^,^^89^ Sweden, ^42^^,^^102^ Australia, ^14^^,^^84^^,^^85^ Brazil, ^19^^,^^50^^,^^77^^,^^83^ and the US. ^37^^,^^43^^,^^49^^,^^52^^,^^81^^,^^82^ There was not much variation in the prevalence estimates among the dentists, dental auxiliaries, and dental students. These observations suggest that all types of dental healthcare providers globally suffer from MSDs due to prolonged static postures. Over three decades, there was no significant change in the trend of MSD, indicating a consistently higher prevalence, highlighting the need to incorporate ergonomics into the dental curriculum.

There was substantial inconsistency in the assessment of prevalence estimates among the studies. The Nordic/standardized Nordic questionnaire was the most commonly used tool to assess MSDs. A few studies used generic questionnaires and single-item questions without adequate validity and reliability. Moreover, the studies used various time recall periods (lifetime, one year, six months, one month, and one week) to assess the prevalence estimates. The studies that used lifetime or extended recall periods might have included pre-existing MSDs that may not be work-related, which could have diluted the estimates of MSD.

MSD can arise from various reasons, and there was a lack of clarity in most of the studies. Only one study explicitly recorded the estimates before and after joining the dental profession. ^58^ There was a general lack of clarity on the estimates reported for various body parts (shoulders, hands, elbow, wrists, legs, ankles, hips, fingers, toes). The studies reported right, left, and bilateral prevalence estimates of MSD without detailing the prevalence for each site. MSD in such areas could have been reported as unilateral and bilateral rather than right, left, and bilateral estimates. Furthermore, there was no uniformity in the evaluation of site-specific assessments among the studies included (e.g. lack of clarity on the terms hand and arms).

The strength of this review is the inclusion of studies that reported the overall estimates of MSD, including many databases, all types of dental healthcare personnel, overall, lifetime and annual estimates, sub-group analysis, gender, and site-specific prevalence estimates. A few limitations were observed in our study. They are the exclusion of studies published in other languages, lack of age-specific prevalence estimates, lack of differentiation between work-related and pre-existing MSDs, causes of MSDs due to inadequate reporting in primary studies, use of self-reported measures of MSD rather than objective measures, and exclusion of studies with no comprehensive assessment or overall estimates of MSD.

The additional confounding factors related to lifestyle (sedentary lifestyle, lack of regular physical exercise, and other extra-curricular activities) could significantly influence the onset and duration of MSD. Furthermore, the number of clinical working days/week, working hours/day, type and duration of procedures, specialization, number of patients/days, remedial measures, and history of MSD in the past could also substantially impact the estimates of MSD. These inconsistencies in the included studies could have influenced the overall prevalence of MSD.

## Conclusions

MSD among dental healthcare personnel is widespread and mostly chronic. Seven out of ten dental healthcare providers could have experienced MSD in the past. However, the severity and self-limiting nature of MSD cannot be underestimated. Awareness, adoption, and maintenance of appropriate ergonomic postures should be encouraged at dental schools and early in the career. Future studies should use the “Strengthening the Reporting of Observational Studies in Epidemiology ( STROBE)” guidelines and use validated questionnaires for reporting MSD.

## Data availability

### Underlying data

Mendeley Data: Underlying data for ‘Musculoskeletal disorders among dental health care professionals’. https://www.doi.org/10.17632/2ttwfmzm9n.2 ^35^

### Reporting guidelines

Mendeley Data: PRISMA checklist for ‘Musculoskeletal disorders among dental health care professionals’. https://www.doi.org/10.17632/2ttwfmzm9n.2 ^35^

Data are available under the terms of the Creative Commons Attribution 4.0 International license (CC-BY 4.0)

## References

[1] Marklund S Mienna CS Wahlström J : Work ability and productivity among dentists: associations with musculoskeletal pain, stress, and sleep. *Int. Arch. Occup. Environ. Health.* 2020;93(2):271–278. 10.1007/s00420-019-01478-5 31654126 PMC7007882

[2] Lindegård A Larsman P Hadzibajramovic E : The influence of perceived stress and musculoskeletal pain on work performance and work ability in Swedish health care workers. *Int. Arch. Occup. Environ. Health.* 2014;87(4):373–379. 10.1007/s00420-013-0875-8 23609321 PMC3996278

[3] Gupta D Devaki M Dommaraju N : Musculoskeletal pain management among dentists: An alternative approach. *Holist. Nurs. Pract.* 2015;29(6):385–390. 10.1097/HNP.0000000000000074 26067590

[4] Pejčić N Petrović V Marković D : Assessment of risk factors and preventive measures and their relations to work-related musculoskeletal pain among dentists. *Work.* 2017;57(4):573–593. 10.3233/WOR-172588 28826201

[5] Shekhawat K Chauhan A Sakthidevi S : Work-related musculoskeletal pain and its self-reported impact among practicing dentists in Puducherry, India. *Indian J. Dent. Res.* 2020;31(3):354–357. 10.4103/ijdr.IJDR_352_18 32769266

[6] Al-Hourani Z Nazzal M Khader Y : Work-related musculoskeletal disorders among Jordanian dental technicians: Prevalence and associated factors. *Work.* 2017;56(4):617–623. 10.3233/WOR-172524 28409763

[7] Taib MFM Bahn S Yun MH : The effects of physical and psychosocial factors and ergonomic conditions on the prevalence of musculoskeletal disorders among dentists in Malaysia. *Work.* 2017;57(2):297–308. 10.3233/WOR-172559 28582951

[8] Kriangkrai R Sirimala N Nathamtong S : Self-reported prevalence and risk factors of musculoskeletal pain in Thai dental students. *Int. Dent. J. Students Res.* 2016;4(3):116–122.

[9] Gupta D Mathur A Patil G : Prevalence of musculoskeletal disorder and alternative medicine therapies among dentists of North India: A descriptive study. *Pharmacognosy Res.* 2015;7(4):350–354. 10.4103/0974-8490.157810 26692749 PMC4660514

[10] Kumar VK Kumar SP Baliga MR : Prevalence of work-related musculoskeletal complaints among dentists in India: A national cross-sectional survey. *Indian J. Dent. Res.* 2013;24(4):428–438. 10.4103/0970-9290.118387 24047834

[11] Hodacova L Sustova Z Cermakova E : Self-reported risk factors related to the most frequent musculoskeletal complaints among Czech dentists. *Ind. Health.* 2015;53(1):48–55. 10.2486/indhealth.2013-0141 25327296 PMC4331194

[12] Lin TH Liu YC Hsieh TY : Prevalence of and risk factors for musculoskeletal complaints among Taiwanese dentists. *J. Dent. Sci.* 2012;7(1):65–71. 10.1016/J.JDS.2012.01.009

[13] Vuletic J Potran M Kalem D : Prevalence and risk factors for musculoskeletal disorders in dentists. *Stomatol. Glas. Srb.* 2013;60(1):24–31. 10.2298/sgs1301024v

[14] Sakzewski L Naser-Ud-Din S : Work-related musculoskeletal disorders in Australian dentists and orthodontists: Risk assessment and prevention. *Work.* 2015;52(3):559–579. 10.3233/WOR-152122 26409367

[15] Khandan M Koohpaei A Shahbazi M : Assessment of Individual and Occupational Risk Factors of Musculoskeletal Disorders Using BPAI among Dentists in Qom, Iran. *Arch. Hyg. Sci.* 2020;9(3):234–245. 10.29252/archhygsci.9.3.234

[16] Hosseini A Choobineh A Razeghi M : Ergonomic Assessment of Exposure to Musculoskeletal Disorders Risk Factors among Dentists of Shiraz, Iran. *J. Dent. Shiraz. Univ. Med. Sci.* 2019;20(1):53–60. 10.30476/dentjods.2019.44564 30937338 PMC6421327

[17] Alnaser MZ Almaqsied AM Alshatti SA : Risk factors for work-related musculoskeletal disorders of dentists in Kuwait and the impact on health and economic status. *Work.* 2021;68(1):213–221. 10.3233/WOR-203369 33427721

[18] Batham C Yasobant S : A risk assessment study on work-related musculoskeletal disorders among dentists in Bhopal, India. *Indian J. Dent. Res.* 2016;27(3):236–241. 10.4103/0970-9290.186243 27411650

[19] Garbin AJÍ Soares GB Arcieri RM : Musculoskeletal disorders and perception of working conditions: A survey of brazilian dentists in São Paulo. *Int. J. Occup. Med. Environ. Health.* 2017;30(3):367–377. 10.13075/ijomeh.1896.00724 28481371

[20] Ylipää V Arnetz BB Preber H : Predictors of good general health, well-being, and musculoskeletal disorders in Swedish dental hygienists. *Acta Odontol. Scand.* 1999;57(5):277–282. 10.1080/000163599428706 10614906

[21] Warren N : Causes of musculoskeletal disorders in dental hygienists and dental hygiene students: A study of combined biomechanical and psychosocial risk factors. *Work.* 2010;35(4):441–454. 10.3233/WOR-2010-0981 20448323

[22] Hashim R Salah A Mayahi F : Prevalence of postural musculoskeletal symptoms among dental students in United Arab Emirates. *BMC Musculoskelet. Disord.* 2021;22(1):30. 10.1186/s12891-020-03887-x 33407336 PMC7788996

[23] Felemban RA Sofi RA Alhebshi SA : Prevalence and predictors of musculoskeletal pain among undergraduate students at a dental school in Saudi Arabia. *Clin. Cosmet. Investig. Dent.* 2021;13:39–46. 10.2147/CCIDE.S292970 33633467 PMC7900777

[24] Alshouibi EN Almansour LA Alqurashi AM : The effect of number of patients treated, dental loupes usage, stress, and exercise on musculoskeletal pain among dentists in Jeddah. *J. Int. Soc. Prev. Community Dent.* 2020;10(3):336–340. 10.4103/jispcd.JISPCD_2_20 32802781 PMC7402259

[25] Chikte UM Khondowe O Louw Q : A meta analysis of the prevalence of spinal pain among dentists. *SADJ.* 2011;66(5):214–218. 23193861

[26] ZakerJafari HR YektaKooshali MH : Work-Related Musculoskeletal Disorders in Iranian Dentists: A Systematic Review and Meta-analysis. *Saf. Health Work.* 2018;9(1):1–9. 10.1016/j.shaw.2017.06.006 30363086 PMC6111132

[27] Hayes MJ Cockrell D Smith DR : A systematic review of musculoskeletal disorders among dental professionals. *Int. J. Dent. Hyg.* 2009;7(3):159–165. 10.1111/j.1601-5037.2009.00395.x 19659711

[28] Shams-Hosseini NS Vahdati T Mohammadzadeh Z : Prevalence of Musculoskeletal Disorders among Dentists in Iran: A Systematic Review. *Mater Sociomed.* 2017;29(4):257–262. 10.5455/MSM.2017.29.257-262 29284995 PMC5723169

[29] Puriene A Janulyte V Musteikyte M : General health of dentists. Literature review. *Stomatologija.* 2007;9(1):10–20. 17449973

[30] Leggat PA Kedjarune U Smith DR : Occupational health problems in modern dentistry: A review. *Ind. Health.* 2007;45(5):611–621. 10.2486/indhealth.45.611 18057804

[31] Lietz J Kozak A Nienhaus A : Prevalence and occupational risk factors of musculoskeletal diseases and pain among dental professionals in Western countries: A systematic literature review and meta-analysis. *PLoS One.* 2018;13(12):e0208628. 10.1371/journal.pone.0208628 30562387 PMC6298693

[32] Pentapati K Chenna D Kumar M : Prevalence of Musculoskeletal Disorders (MSD) among Dental Health Care Workers. 2021. 10.37766/inplasy2021.5.0100

[33] Ouzzani M Hammady H Fedorowicz Z : Rayyan-a web and mobile app for systematic reviews. *Syst. Rev.* 2016;5(1):210. 10.1186/s13643-016-0384-4 27919275 PMC5139140

[34] Hoy D Brooks P Woolf A : Assessing risk of bias in prevalence studies: modification of an existing tool and evidence of interrater agreement. *J. Clin. Epidemiol.* 2012;65(9):934–939. 10.1016/j.jclinepi.2011.11.014 22742910

[35] Pentapati K Deepika C : Musculoskeletal disorders among dental health care professionals. 2022;1. 10.17632/2TTWFMZM9N.1

[36] Akar GC Aksoy G Özmutaf NM : An assessment of awareness and self-report about occupation-related health problems among dental laboratory technicians in Turkey. *Nobel Med.* 2009;5(3):27–32.

[37] Lalumandier JA McPhee SD Parrott CB : Musculoskeletal pain: prevalence, prevention, and differences among dental office personnel. *Gen. Dent.* 2001;49(2):160–166. 12004695

[38] Gowri Sankar S Reddy PV Reddy BR : The Prevalence of Work-related Musculoskeletal Disorders among Indian Orthodontists. *J. Indian Orthod. Soc.* 2012;46(4):264–268. 10.5005/jp-journals-10021-1102

[39] Sustová Z Hodacová L Kapitán M : The prevalence of musculoskeletal disorders among dentists in the Czech Republic. *Acta Med. (Hradec Kralove).* 2013;56(4):150–156. 10.14712/18059694.2014.10 24693796

[40] Kurşun Ş Evirgen S Akbulut N : Work characteristics and musculoskeletal disorders among postgraduate dental students: A pilot study. *J. Musculoskelet. Pain.* 2014;22(1):62–67. 10.3109/10582452.2014.883010

[41] El-Naji W Al Warawreh AM Al-Saraireh SA : Occupational hazards among Jordanian dentists. *Pakistan Oral Dent J.* 2019;39(2):129.

[42] Åkesson I Johnsson B Rylander L : Musculoskeletal disorders among female dental personnel - Clinical examination and a 5-year follow-up study of symptoms. *Int. Arch. Occup. Environ. Health.* 1999;72(6):395–403. 10.1007/s004200050391 10473839

[43] Anton D Rosecrance J Merlino L : Prevalence of musculoskeletal symptoms and carpal tunnel syndrome among dental hygienists. *Am. J. Ind. Med.* 2002;42(3):248–257. 10.1002/ajim.10110 12210693

[44] Szymańska J : Disorders of the musculoskeletal system among dentists from the aspect of ergonomics and prophylaxis. *Ann. Agric. Environ. Med.* 2002;9(2):169–173. 12498585

[45] Polat Z Başkan S Altun S : Musculoskeletal symptoms of dentists from south-east turkey. *Biotechnol. Biotechnol. Equip.* 2007;21(1):86–90. 10.1080/13102818.2007.10817421

[46] Dajpratham P Ploypetch T Kiattavorncharoen S : Prevalence and associated factors of musculoskeletal pain among the dental personnel in a dental school. *J. Med. Assoc. Thail.* 2010;93(6):714–721. 20572377

[47] Kierklo A Kobus A Jaworska M : Work-related musculoskeletal disorders among dentists - A questionnaire survey. *Ann. Agric. Environ. Med.* 2011;18(1):79–84. 21736272

[48] Tirgar A Javanshir K Talebian A : Musculoskeletal disorders among a group of Iranian general dental practitioners. *J. Back Musculoskelet. Rehabil.* 2015;28(4):755–759. 10.3233/BMR-140579 25547232

[49] Humann P Rowe DJ : Relationship of Musculoskeletal Disorder Pain to Patterns of Clinical Care in California Dental Hygienists. *J. Dent. Hyg. JDH.* 2015;89(5):305–312. 26519494

[50] Freire AC d GF Soares GB Rovida TAS : Musculoskeletal disorders among dentists in northwest area of the state of São Paulo, Brazil. *Brazilian J. Oral Sci.* 2016;15(3):190–195. 10.20396/bjos.v15i3.8649979

[51] Hegde S Donly A Shankar K : Prevalence of Musculoskeletal Disorders among Dental Professionals-A Questionnaire Study. *Indian J. Public Heal. Res. Dev.* 2018;9(3):33–37. 10.5958/0976-5506.2018.00178.X

[52] Pope-Ford R Pope-Ozimba J : Musculoskeletal disorders and emergent themes of psychosocial factors and their impact on health in dentistry. *Work.* 2020;65(3):563–571. 10.3233/WOR-203110 32116274

[53] Ohlendorf D Naser A Haas Y : Prevalence of musculoskeletal disorders among dentists and dental students in germany. *Int. J. Environ. Res. Public Health.* 2020;17(23):1–19. 10.3390/ijerph17238740 PMC7727829 33255491

[54] Ohlendorf D Haas Y Naser A : Prevalence of Muscular Skeletal Disorders among Qualified Dental Assistants. *Int. J. Environ. Res. Public Health.* 2020;17(10):3490. 10.3390/ijerph17103490 32429484 PMC7277800

[55] Alpa V Ramdev N Parekh V : A survey on prevalence of work related musculoskeletal disorder among the dentists in Vadodara city - a questionnaire based study. *J. Pearldent.* 2014;5(1):31–36.

[56] Mendegeri V Ramdurg PK Kambale S : Prevalence Of Musculoskeletal Disorders Among Dentists: A Pilot Study. *Indian J. Dent. Sci.* 2014;6(5):16–20.

[57] Kanaparthy A Kanaparthy R Boreak N : Postural awareness among dental students in Jizan, Saudi Arabia. *J. Int. Soc. Prev. Community Dent.* 2015;5(Suppl 2):S107–S111. 10.4103/2231-0762.172950 26942113 PMC4756563

[58] Alghadir A Zafar H Iqbal ZA : Work-related musculoskeletal disorders among dental professionals in Saudi Arabia. *J. Phys. Ther. Sci.* 2015;27(4):1107–1112. 10.1589/jpts.27.1107 25995567 PMC4433988

[59] Bhagwat S Hegde S Mandke L : Prevalence of musculoskeletal disorders among Indian dentists: A pilot survey with assessment by rapid entire body assessment. *World J. Dent.* 2015;6(1):39–44. 10.5005/jp-journals-10015-1310

[60] Revankar V Chakravarthy Y Naveen S : Musculoskeletal disorders and mental health-related issues as occupational hazards among dental practitioners in Salem city: A cross-sectional study. *J. Pharm. Bioallied Sci.* 2017;9(5):S228–S230. 10.4103/jpbs.JPBS_145_17 29284969 PMC5731018

[61] Coskun Benlidayi I Al-Bayati Z Guzel R : Neither got a good bill of musculoskeletal health: a comparative study among medical and dental students. *Acta Clin. Belgica Int. J. Clin. Lab. Med.* 2019;74(2):110–114. 10.1080/17843286.2018.1483564 29874980

[62] Zafar H Almosa N : Prevalence of work-related musculoskeletal disorders among dental students of King Saud University, Riyadh, Kingdom of Saudi Arabia. *J. Contemp. Dent. Pract.* 2019;20(4):449–453. 10.5005/jp-journals-10024-2537 31308275

[63] Harris ML Sentner SM Doucette HJ : Musculoskeletal disorders among dental hygienists in Canada. *Can. J. Dent. Hyg.* 2020;54(2):61–67. 33240365 PMC7668274

[64] Uppada UK Susmitha M Ullah Hussaini S : Ergonomics among dentists in the states of Telangana and Andhra Pradesh. *Natl. J. Maxillofac. Surg.* 2020;11(2):253–257. 10.4103/njms.NJMS_33_20 33897190 PMC8051662

[65] Uppada UK Sinha R Madishetti S : Ergonomics among oral and maxillofacial surgeons in the Indian States of Telangana and Andhra Pradesh - An evaluative study. *Ann. Maxillofac. Surg.* 2020;10(2):325–329. 10.4103/ams.ams_39_20 33708575 PMC7944000

[66] Movahhed T Ajami B Soltani M : Musculoskeletal pain reports among Mashhad dental students, Iran. *Pak. J. Biol. Sci.* 2013;16(2):80–85. 10.3923/pjbs.2013.80.85 24199491

[67] Gandolfi MG Zamparini F Spinelli A : Musculoskeletal disorders among italian dentists and dental hygienists. *Int. J. Environ. Res. Public Health.* 2021;18(5):1–20. 10.3390/ijerph18052705 33800193 PMC7967428

[68] Ayers KMS Thomson WM Newton JT : Self-reported occupational health of general dental practitioners. *Occup. Med. (Chic Ill).* 2009;59(3):142–148. 10.1093/occmed/kqp004 19223433

[69] Muralidharan D Fareed N Shanthi M : Musculoskeletal Disorders among Dental Practitioners: Does It Affect Practice? *Epidemiol. Res. Int.* 2013;2013:1–6. 10.1155/2013/716897

[70] Kazancioglu HO Bereket MC Ezirganli S : Musculoskeletal complaints among oral and maxillofacial surgeons and dentists: A questionnaire study. *Acta Odontol. Scand.* 2013;71(3-4):469–474. 10.3109/00016357.2012.696688 23317089

[71] Rafeemanesh E Jafari Z Kashani FO : A study on job postures and musculoskeletal illnesses in dentists. *Int. J. Occup. Med. Environ. Health.* 2013;26(4):615–620. 10.2478/s13382-013-0133-z 24142742

[72] Zarra T Lambrianidis T : Musculoskeletal disorders amongst Greek endodontists: A national questionnaire survey. *Int. Endod. J.* 2014;47(8):791–801. 10.1111/iej.12219 24283200

[73] Aljanakh M Shaikh S Siddiqui AA : Prevalence of musculoskeletal disorders among dentists in the Ha’il Region of Saudi Arabia. *Ann. Saudi Med.* 2015;35(6):456–461. 10.5144/0256-4947.2015.456 26657230 PMC6074478

[74] Tamo T Kalita C Bhuyan A : Evaluation of occupational musculoskeletal disorders and related risk factors among dentists working in North East India. *Dent. Med. Res.* 2015;3(2):43. 10.4103/2348-1471.159182

[75] Sahu D Tandon S Dhingra S : Prevalence of musculoskeletal disorders among dentists: A pilot cross-sectional survey. *J. Indian Assoc. Public Heal. Dent.* 2015;13(3):307. 10.4103/2319-5932.165281

[76] Rayyan M Hetou S Al Salem R : Work-related Musculoskeletal Disorders among Dental Students of Different Academic Levels. *J. Int. Oral Heal.* 2016;8(4):471–475. 10.2047/jioh-08-04-12

[77] Santos RR dos Garbin CAS Batista JA : Prevalence of musculoskeletal pain in dental students and associated factors. *Brazilian J. Oral Sci.* 2019;18:e191668. 10.20396/bjos.v18i0.8657270

[78] Gandham A Boppana N Vinnakota N : Assessment of musculoskeletal disorders and associated risk factors among dentists in Rajahmundry City: A cross-sectional study. *J. Indian Assoc. Public Heal. Dent.* 2019;17(2):114. 10.4103/jiaphd.jiaphd_9_19

[79] Netanely S Luria S Langer D : Musculoskeletal disorders among dental hygienist and students of dental hygiene. *Int. J. Dent. Hyg.* 2020;18(2):210–216. 10.1111/idh.12428 32012436

[80] Berdouses E Sifakaki M Katsantoni A : Work-Related Musculoskeletal Disorders among Greek Dentists - A Nationwide Survey. *Dent. Res. Oral Heal.* 2020;3(4):169–181.

[81] Osborn JB Newell KJ Rudney JD : Musculoskeletal pain among Minnesota dental hygienists. *J. Dent. Hyg. JDH/Am. Dent. Hyg. Assoc.* 1990;64(3):132–138. 2149148

[82] Barry RM Spolarich AE Weber M : Impact of Operator Positioning on Musculoskeletal Disorders and Work Habits Among Mississippi Dental Hygienists. *J. Dent. Hyg. JDH.* 2017;91(6):6–14. 29378801

[83] Carvalho MVD de Soriano EP França Caldas A de : Work-Related Musculoskeletal Disorders Among Brazilian Dental Students. *J. Dent. Educ.* 2009;73(5):624–630. 10.1002/j.0022-0337.2009.73.5.tb04737.x 19433537

[84] Marshall ED Duncombe LM Robinson RQ : Musculoskeletal symptoms in New South Wales dentists. *Aust. Dent. J.* 1997;42(4):240–246. 10.1111/j.1834-7819.1997.tb00128.x 9316311

[85] Leggat PA Smith DR : Musculoskeletal disorders self-reported by dentists in Queensland, Australia. *Aust. Dent. J.* 2006;51(4):324–327. 10.1111/j.1834-7819.2006.tb00451.x 17256307

[86] Ellapen TJ Narsigan S Herdeen HJ van : Impact of poor dental ergonomical practice. *SADJ.* 2011;66(6):272, 274–272, 277. 23198475

[87] Tezel A Kavrut F Tezel A : Musculoskeletal disorders in left- and right-handed Turkish dental students. *Int. J. Neurosci.* 2005;115(2):255–266. 10.1080/00207450590519517 15764005

[88] Moradia S Patel P : A Study on Occupational Pain among Dentists of Surat City. *Natl J Community Med.* 2011;2(1):116–118.

[89] Shadmehr A Haddad O Azarnia S : Disorders of the musculoskeletal system among Tehran, Iranian dentists. *J Musculoskelet Pain.* 2014;22(3):256–259. 10.3109/10582452.2014.883022

[90] Rehman B Aslam A Afsheen A : Ergonomic hazards to dental surgeons: A cross-sectional study. *Pakistan Oral Dent J.* 2016;39(2):129–132.

[91] Phedy P Gatam L : Prevalence and associated factors of musculoskeletal disorders among young dentists in Indonesia. *Malaysian Orthop J.* 2016;10(2):1–5. 10.5704/MOJ.1607.001 28435553 PMC5333646

[92] Cho K Young CH Han GS : Risk factors associated with musculoskeletal symptoms in Korean dental practitioners. *J. Phys. Ther. Sci.* 2016;28(1):56–62. 10.1589/jpts.28.56 26957728 PMC4755974

[93] Al-Rawi NH El Khatib H Rajoub L : Work-related musculoskeletal pain among different dental specialists in United Arab Emirates. *J. Contemp. Dent. Pract.* 2016;17(8):639–644. 10.5005/jp-journals-10024-1904 27659080

[94] Meisha DE Alsharqawi NS Samarah AA : Prevalence of work-related musculoskeletal disorders and ergonomic practice among dentists in Jeddah, Saudi Arabia. *Clin. Cosmet. Investig. Dent.* 2019;11:171–179. 10.2147/CCIDE.S204433 31308760 PMC6615716

[95] Rahman N Adnan M Yusoff A : Work-related musculoskeletal symptoms and coping strategies among dental auxiliaries at hospital universiti Sains Malaysia. *Indian J. Dent. Res.* 2020;31(1):61–66. 10.4103/ijdr.IJDR_430_18 32246684

[96] Aboalshamat Aboalshamat KT Aboalshamat KT : Nordic assessment of occupational disorders among dental students and dentists in Saudi Arabia. *J. Int. Soc. Prev. Community Dent.* 2020;10(5):561–568. 10.4103/JISPCD.JISPCD_142_20 33282764 PMC7685269

[97] Kumar M Pai KM Vineetha R : Occupation-related musculoskeletal disorders among dental professionals. *Med. Pharm. Reports.* 2020;93(4):405–409. 10.15386/MPR-1581 33225267 PMC7664727

[98] Ahmad NS Abdullah AAA Thyng OK : Musculoskeletal Disorders Among Dental Students. *J. Res. Med. Dent. Sci.* 2020;8(3):32–38.

[99] Bhuvaneshwari S Shveta J Kaur J : Assessment of Various Dental Occupational Hazards and Safety Measures among Dentists of Odisha, India. *J. Contemp. Dent. Pract.* 2021;21(10):1165–1169. 10.5005/JP-JOURNALS-10024-2885 33686041

[100] Senosy SA Anwar MM Elareed HR : Profession-related musculoskeletal disorders among Egyptian physicians and dentists. *J. Public Heal.* 2020;28(1):17–22. 10.1007/s10389-019-01016-0

[101] Puriene A Aleksejuniene J Petrauskiene J : Self-reported occupational health issues among Lithuanian dentists. *Ind. Health.* 2008;46(4):369–374. 10.2486/indhealth.46.369 18716385

[102] Rundcrantz BL Johnsson B Moritz U : Pain and discomfort in the musculoskeletal system among dentists. A prospective study. *Swed. Dent. J.* 1991;15(5):219–228. 1837389

[103] Kerosuo E Kerosuo H Kanerva L : Self-reported health complaints among general dental practitioners, orthodontists, and office employees. *Acta Odontol. Scand.* 2000;58(5):207–212. 10.1080/000163500750051755 11144871

[104] Zoidaki A Riza E Kastania A : Musculoskeletal disorders among dentists in the Greater Athens area, Greece: risk factors and correlations. *J. Public Health (Bangkok).* 2013;21(21):163–173. 10.1007/S10389-012-0534-7

[105] Šćepanović D Klavs T Verdenik I : The Prevalence of Musculoskeletal Pain of Dental Workers Employed in Slovenia. *Work Heal. Saf.* 2019;67(9):461–469. 10.1177/2165079919848137 31288626

