# Supplementary material for: Prevalence of musculoskeletal disorders among dental healthcare providers: A systematic review and meta-analysis
Source: F1000Res. 2022 Dec 12;11:1062. Originally published 2022 Sep 16. [Version 2] doi: 10.12688/f1000research.124904.2 (PMC9709350; doi:10.12688/f1000research.124904.2)
Supplement: Peer review history

# Peer review: Reviewer response for version 1

**DOI:** 10.5256/f1000research.137145.r151602

**Published:** 2022-11-29

**Authors:** Preethi Balan

**Affiliations:** 1 Singapore Oral Microbiomics Initiative, National Dental Research Institute Singapore, National Dental Center, Singapore, Singapore

The authors have studied the prevalence estimates of MSD among dental healthcare workers using a systematic search of five databases. The authors observed high prevalence of MSD among dental healthcare workers which highlights the importance of them becoming aware of and adopting appropriate ergonomic postures early in their careers in order to reduce work-related MSD.

 The paper is well written and covers most of the aspects in this issue. There are a few suggestions below:

  

1. Were the two reviewers calibrated before doing the search? If possible the authors can provide the kappa statistics for agreement between the two reviewers.
2. The authors can provide the numbers of hits with each of the five databases in the prisma flowchart.
3. The authors can provide the years included in the search. Also if there was any manual search carried out in addition to the automated search.
4. Please elaborate in the inclusion/exclusion criteria regarding the type of studies considered, e.g. randomized and controlled clinical trials/observational studies/case control studies etc.

Are the rationale for, and objectives of, the Systematic Review clearly stated?

Yes

Is the statistical analysis and its interpretation appropriate?

Yes

Are sufficient details of the methods and analysis provided to allow replication by others?

Partly

Are the conclusions drawn adequately supported by the results presented in the review?

Yes

Reviewer Expertise:

Clinical oral health research

I confirm that I have read this submission and believe that I have an appropriate level of expertise to confirm that it is of an acceptable scientific standard.

# Peer review: Reviewer response for version 1

**DOI:** 10.5256/f1000research.137145.r151604

**Published:** 2022-10-13

**Authors:** Athira Nandakumar

**Affiliations:** 1 Department of Epidemiology and Preventive Medicine, Kagoshima University Graduate School of Medical and Dental Sciences, Kagoshima, Japan

**Introduction:**

 The authors showed the high prevalence of Musculoskeletal disorder (MSD) among dental health care providers in a worldwide scenario. MSD-related problems seem to be a significant issue for this profession and this is an already known problem. The authors tried to put up possibly all available publications related to MSD and did this attempt. However, certain changes in the manuscript and corrections to spelling mistakes or typos are recommended.

 The exact definition of MSD is missing.

**Methods:**

1) Inclusion Criteria:

**Question:** Is there any previously published systematic review included in this study?

**Question: **Hygienists mentioned here are missing in Table 2. Are they included in auxiliaries?

**Question: **What kind of studies were included - Cross-sectional/cohort/case-control?

**Question: **Were there any self-reported studies?

2) RoB:

**Question: **What is the cutoff point used in this study? It is not mentioned that some cut-off point was used here.

**Question:** How many articles were excluded after RoB calculations?

3) Results:

**Question: **Meta-analysis was performed for 89? Or 88?

**Question: **What is the reason for the exclusion of 146 publications after the full screening of the full text?

4) Prevalence: There is a repeated word “than than”.

5) Geographic distribution:

**Question: **The authors mentioned that Australia (4), Africa (1), and north and south America (7,4) why not Asia and Europe (N=?)?

 It may be better to revise this “One study from Africa (N=1)".

6) Figure 1:

**Question: **Where is the N=89 in this chart?

**Question: **What are the other sources used to identify additional records?

7) Table 2:

**Question: **Why is Africa not mentioned here?

**Question: **Malaysia has the highest estimates. What is the reason? Is it due to the inappropriate definition of MSD in Malaysia?

**Question: **The estimate for one year (0.82) is more than for a lifetime (0.78); What may be the possible reason?

**Question: **What is the definition of “mixed”?

**Question: **Dental auxiliaries seem to have more difficulties with MSD with an estimate of 0.83 (0.69-0.97). Is it not because 2-3 categories are grouped together?

8) Limitation:

**Question: **It’s mentioned that “the exclusion of studies published in other languages”; does it mean other than English?

**Discussion:**

**Question: **What was the national survey estimate? Mentioned in references 37,80? Better to mention Greek and Czech surveys.

**Question: **Reference 37, is it a national survey?

**Question: **What was the prevalence of the studies that used the Nordic questionnaire?

Are the rationale for, and objectives of, the Systematic Review clearly stated?

Yes

Is the statistical analysis and its interpretation appropriate?

Yes

Are sufficient details of the methods and analysis provided to allow replication by others?

Partly

Are the conclusions drawn adequately supported by the results presented in the review?

Yes

Reviewer Expertise:

Epidemiologist

I confirm that I have read this submission and believe that I have an appropriate level of expertise to confirm that it is of an acceptable scientific standard.

# Response

**Published:** 2022-10-19

**Authors:** Kalyana Pentapati

**Affiliations:** Manipal Academy of Higher Education, India

We thank the reviewer for their effort in reviewing this manuscript. 

 Please find responses for the queries. The changes that are required to be done in the manuscript will be incorporated. 

 Introduction:

 The authors showed the high prevalence of Musculoskeletal disorder (MSD) among dental health care providers in a worldwide scenario. MSD-related problems seem to be a significant issue for this profession and this is an already known problem. The authors tried to put up possibly all available publications related to MSD and did this attempt. However, certain changes in the manuscript and corrections to spelling mistakes or typos are recommended.

 The exact definition of MSD is missing.

**Response:** MSDs are defined as musculoskeletal system and connective tissue diseases and disorders when the event or exposure leading to the case is bodily reaction (e.g., bending, climbing, crawling, reaching, twisting), overexertion, or repetitive motion. MSDs do not include disorders caused by slips, trips, falls, or similar incidents (Bureau of Labor Statistics of the Department of Labor. NIOSH workers health chartbook 2004. NIOSH Publication No. 2004-146. Washington, D.C). We will add the same into the mansucript. 

 Methods:

 1) Inclusion Criteria:

 Question: Is there any previously published systematic review included in this study?

**Response: **No

 Question: Hygienists mentioned here are missing in Table 2. Are they included in auxiliaries?

**Response: **Yes

 Question: What kind of studies were included - Cross-sectional/cohort/case-control?

**Response:** We included studies from which prevalence could be calculated. However, most of the included studies were cross-sectional (n=86)

 Question: Were there any self-reported studies?

**Response: **All the studies were self-reported

 2) RoB:

 Question: What is the cutoff point used in this study? It is not mentioned that some cut-off point was used here.

**Response:** Each question has two levels, low risk (0) and high risk (1). The total of all nine questions was used to categorize the studies as “low (0–3), moderate (4–6), or high risk (7–9)”. (This information was in the risk of bias assessment in the methodology section)

 Question: How many articles were excluded after RoB calculations?

**Response: **Nil. Sensitivity analysis did not show any change in the overall estimate

 3) Results:

 Question: Meta-analysis was performed for 89? Or 88?

**Response: **Meta-analysis was performed for 89 estimates that yielded from 88 publications.

 Question: What is the reason for the exclusion of 146 publications after the full screening of the full text?

**Response:** During the full-text screening of the publications, we excluded studies which had unclear outcome (n=138), inappropriate study design (n=4), short communications (n=2), and secondary analysis (n=2). This information is presented in figure 1.

 4) Prevalence: There is a repeated word “than than”.

**Response:** Thank you. We will incorporate the change.

 5) Geographic distribution:

 Question: The authors mentioned that Australia (4), Africa (1), and north and south America (7,4) why not Asia and Europe (N=?)?

**Response: **We didn’t want to replicate the data that has been presented in tables. Continent and country wise estimates with number of studies were presented in table 2.

 It may be better to revise this “One study from Africa (N=1)".

 Response: We will incorporate the change.

 6) Figure 1:

 Question: Where is the N=89 in this chart?

**Response:** 89 estimates were obtained from 88 publications. In the flow chart we have highlighted the process flow with respect to the number of publications.

 Question: What are the other sources used to identify additional records?

**Responses: **We have sought additional publications from the reference list given at the end of each article.

 7) Table 2:

 Question: Why is Africa not mentioned here?

**Response:** As there was only one study reported from Africa, we could not pool the estimate.

 Question: Malaysia has the highest estimates. What is the reason? Is it due to the inappropriate definition of MSD in Malaysia?

**Response:** We could not identify the reason for this. Many factors like the number of clinical working days/week, working hours/day, type and duration of procedures, specialization, number of patients/days, remedial measures, and history of MSD in the past can have substantially impact on the estimates of MSD.

 Question: The estimate for one year (0.82) is more than for a lifetime (0.78); What may be the possible reason?

**Response:** Different studies have used different time frames to report the MSD. Most studies have reported one year prevalence (n=65) followed by lifetime prevalence. Some studies reported both. Hence, it is not possible to substantiate what could be reason of this disparity. There could be possibility of recall bias among the participants that report lifetime events when compared to one-year events.  

 Question: What is the definition of “mixed”?

**Response: **Studies that have not reported estimates separately for dentists, dental students or dental auxiliaries or studies that included different type of dental personnel.

 Question: Dental auxiliaries seem to have more difficulties with MSD with an estimate of 0.83 (0.69-0.97). Is it not because 2-3 categories are grouped together?

**Response: **Among the ten publications that were included in the dental auxiliaries, 6 studies were among dental hygienists (prevalence: 80.42 to 98.36), two were among dental technicians (prevalence 23.8 and 100%) and one was among dental assistant (prevalence 81.71%) and one was among dental auxiliaries (prevalence 81.7). Hence, estimates are high and may not related to the categorisation.

 8) Limitation:

 Question: It’s mentioned that “the exclusion of studies published in other languages”; does it mean other than English?

**Response: **Yes

 Discussion:

 Question: What was the national survey estimate? Mentioned in references 37,80? Better to mention Greek and Czech surveys.

**Response: **Yes, we will incorporate the change.

 Question: Reference 37, is it a national survey?

**Response: **No. It is a study among dental personnel in US dental army.

 Question: What was the prevalence of the studies that used the Nordic questionnaire?

**Response: **45 studies used Nordic questionnaire.
